# Panniculitis caused by progesterone injection can be treated by physical therapy

**DOI:** 10.1111/dth.14501

**Published:** 2020-11-12

**Authors:** Weizhu Xiao, Xiuxi Huang, Cuifen Lin, Yanying Liu, Shuping Chen, Ruiyun Wu

**Affiliations:** ^1^ The Second Affiliated Hospital of Fujian Medical University Fujian China

**Keywords:** progesterone, panniculitis, physicotherapeutics, skin temperature, subcutaneous injection

## Abstract

A method for the treatment of panniculitis caused by progesterone injection is introduced. Sixteen patients achieved good results. This is a 9‐year single center retrospective study. Of all the 5633 patients who received progesterone injection, 16 developed panniculitis at the injection site. Pathological examination confirmed the occurrence of panniculitis. The patient received physical therapy. These treatments are determined by the course of the patient. Compared with patients without panniculitis, patients with panniculitis received more than one injection of progesterone. In 16 patients, symptoms and local signs disappeared completely in 15 patients. One patient did not take physical therapy according to the doctor's advice after the treatment improved. However, 1 month later, the patient went to see the doctor again and received the relevant physical therapy, and still achieved good results. Progesterone injection may lead to panniculitis, which is rare but may cause serious consequences. Physical therapy can be effective.

## 1 BACKGROUND

Panniculitis refers to the nonsuppurative inflammation caused by the stimulation of nonautologous substances in subcutaneous fat due to external factors. Progesterone injection is an important part of assisted reproductive technology. This treatment may lead to the development of lipid membrane inflammation.^1^ There is no specific treatment for the disease.^2^ For these patients, physical therapy may be a good option. The authors report on the treatment of patients with liposembranitis following progesterone injection in a large hospital in the past 9 years, preliminarily confirming the efficacy of physical therapy.

## 2 METHODS

Patients who received progesterone injections at a large hospital from January 2011 to December 2019 were included in the study. This is a retrospective study. The inclusion criteria and exclusion criteria are shown in Table 1. The authors obtained general information of these patients through the case system. The data included the patient's age, body mass index, pregnancy status, progesterone dosage, Visual Analogue scale score at visit, etc. Progesterone injection additives are obtained by reading the instructions. The use of progesterone injection with different adjuvants depends on the procurement of hospitals at different times. The number and dose of progesterone injections the patients received were also recorded. Institutional review board approval was received from the Second Affiliated Hospital of Fujian Medical University.

**TABLE 1:** Exclusion criteria and inclusion criteria

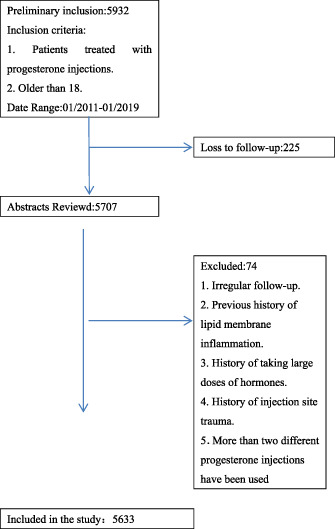

The main symptom in these patients is pain, which worsens during the night. Pain is characterized by needle‐like tingling. Changes in posture can cause pain to worsen. The swelling will not be confined to or around the injection site. The swelling and pain of several patients spread to the lower back, perineum, medial thigh and lateral thigh. The patient's sleep was severely affected before the visit. Patients considered likely to have liposemembranitis were the first to undergo a local puncture. The purpose of this procedure is to obtain pathological specimens for the diagnosis of the patient. The patient also underwent ultrasound and blood tests. Blood tests include human chorionic gonadotropin (HCG), white blood cells and C‐reactive protein (CRP). The patient received physical therapy before the pathology came out.

The patient received physical therapy before the pathology came out. In the early stages of the disease, the site of the disease is sterilized by the nurse with chlorhexidine. The main ingredient of chlorhexidine is dichlorophenbiguanide hexane.^3^ This medicine has quite strong broad spectrum antibacterial, bactericidal action, is a better sterilization and disinfectant. When partially dried, SANYRENE is applied to the affected area. SANYRENE is a liquid dressing consisting of corn peroxide oil and a small amount of anise. This product can be used on the skin surface for pressure ulcers, dry skin and areas of risk. The purpose of using this product is to protect the wound.^4^ The Mesalt dressing was moistened with iodophor and subsequently covered over the affected area. Mesalt consists of a soft nonwoven material filled with sodium chloride. This product can be used for the absorption of necrotic tissue and exudate from moderate to severe noninfected/infected wounds.^5^ We use this dressing to absorb the exudate of dead tissue. The Mepilex dressing is covered on the outermost layer. Mepilex can be used for wound protection and exudate absorption.^6^ An elastic bandage is used to fasten the dressing. The Mesalt dressing can be replaced by a 50% magnesium sulfate patch after the patient's symptoms are relieved and the swelling of the affected area has decreased by more than 50%. There is no interest relationship between the author and the manufacturer of the therapeutic product.

### 2.1 Statistical analysis

SPSS 20.0 was used to process the data. Independent sample *t* test and group *t* test are used to analyze count data. Chi‐square test is used to analyze measurement data. When *P* < .05, the difference was considered to be statistically significant.

## 3 RESULTS

A total of 5633 patients were included in the study. Of these, 16 patients developed lipid membrane inflammation. There were no statistically significant differences in age, body mass index, pain duration or Visual Analogue scale score between the two groups. Most patients with liposemembranitis received progesterone injections supplemented with medium chain oil. This difference was statistically significant when compared with patients without lipid inflammation. Patients who developed lipmitis received more progesterone injections than those who did not, and the difference was statistically significant. There was no statistically significant difference in HCG between the two groups at the time of initial consultation. However, patients with lipid membrane inflammation had higher leucocyte level and CRP level, and the difference was statistically significant. There was no statistically significant difference in pregnancy outcomes between the two groups at the time of visit. Similarly, there was no statistically significant difference in pregnancy outcomes between the two groups. The above data can be reflected in Table 2.

**TABLE 2:** Comparison of clinical data between the two groups

Variable	Patients with panniculitis (n = 16)	Patients without panniculitisp (n = 5617)	Statistic (*t* value/χ^2^‐value)	*P*‐value
Progesterone adjuvant			23.53	0.00
Vegetable oil	1	3620		
Mineral oil	15	1997		
The number of progesterone injections	5.13 ± 1.25	3.16 ± 1.97	3.99	<0.01
Age (years)	23.51 ± 3.17	25.22 ± 5.69	1.20	0.23
BMI	27.52 ± 1.05	26.95 ± 2.33	0.98	0.33
Duration of pain (days)	11.56 ± 3.15	13.15 ± 6.72	0.95	0.34
VAS score at visit	5.55 ± 0.35	6.12 ± 2.11	1.09	0.28
HCG (IU/mL)	35 156.23 ± 2356.13	32 563.38 ± 6785.61	1.53	0.13
White blood cell (10^9^/L)	10.13 ± 2.63	7.96 ± 3.26	2.66	0.01
CRP (mg/L)	26.57 ± 3.65	6.25 ± 2.13	38.00	<0.01
Pregnancies			0.20	0.65
Singletons	16	5547		
Multiplets	0	70		
Pregnancy outcome			0.01	0.90
Pregnancy success	15	5223		
Abortion	1	394		

Abbreviations: BMI, body mass index; CRP, C‐reactive protein; HCG, human chorionic gonadotropin; VAS, Visual Analogue scale.

**FIGURE 1:**
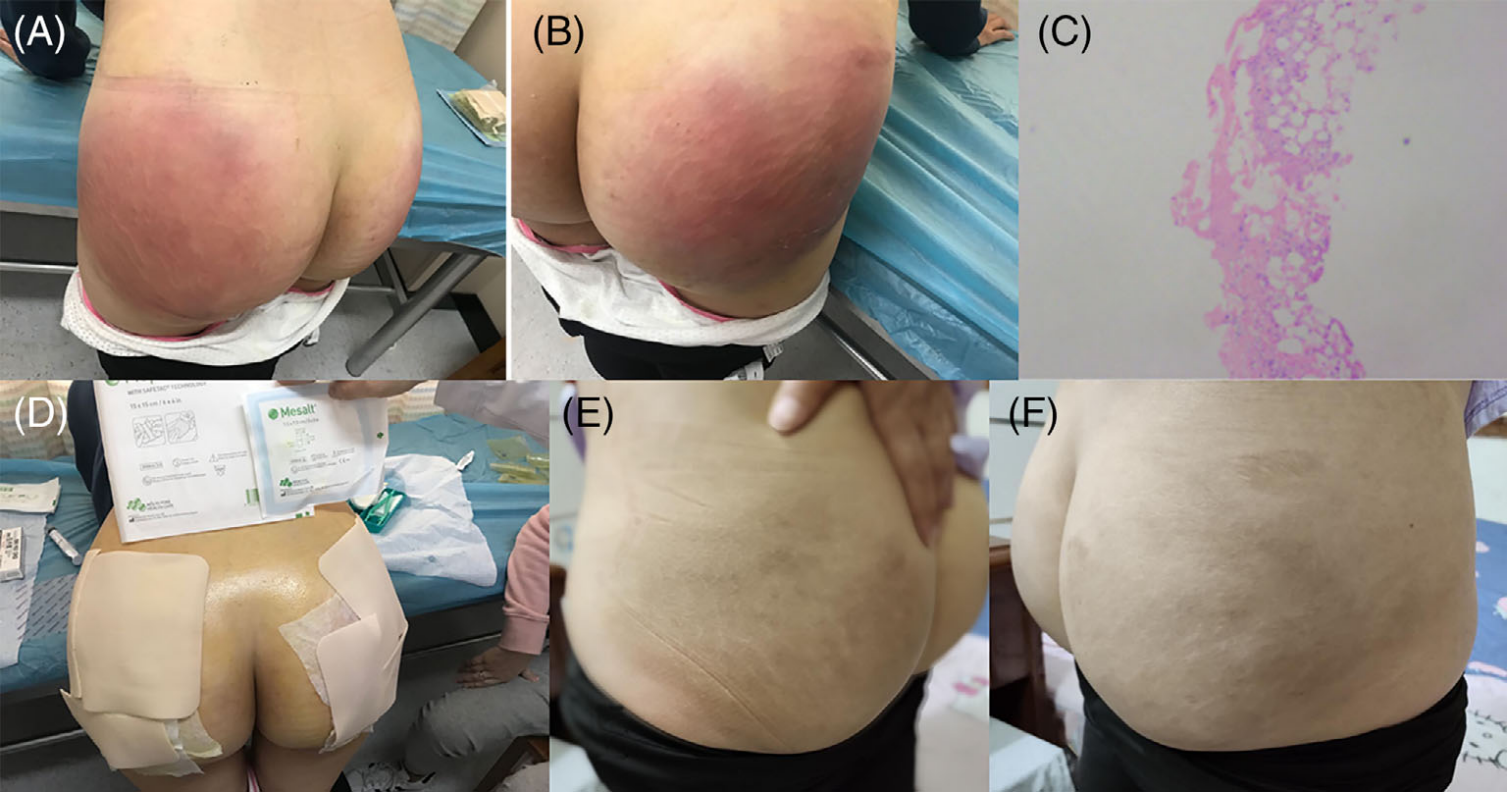
A, Pain and swelling of the left hip occurred after progesterone injection. B, The same symptoms appear in the right buttock. C, The occurrence of panniculitis was confirmed by pathology. D, The patient received physical therapy. E, The left hip recovered well. F, The right hip recovered well

With the increase of treatment times, the local symptoms of patients gradually relieved until disappeared. There was a positive correlation between the skin temperature and the degree of pain. Two to 3 days after treatment, the skin temperature of the affected area will drop significantly. Three patients still had residual induration after treatment, but no swelling and pain symptoms. One patient did not take physical therapy according to the doctor's advice after the treatment improved. However, 1 month later, the patient went to see the doctor again and received the relevant physical therapy, and still achieved good results. The treatment process is shown in Figure 1.

## 4 DISCUSSION

Progesterone is a natural progesterone, which is considered to be the most safe and effective exogenous progesterone supplement.^7^ This drug can maintain decidual endometrium, relax uterine smooth muscle, improve uterine blood supply and immune regulation in early pregnancy.^8^ The drug has been widely used in prevention and treatment of abortion and progesterone supplement related to assisted reproductive technology, and achieved good results.^9^ Intramuscular injection of progesterone for patients is a very common treatment, especially for patients with luteal insufficiency. This treatment may cause some complications, the most common being local pain and induration. Among all the complications, panniculitis is very rare but difficult to treat. Adipocytes are fragile. The injury caused by needle puncture is easy to cause subcutaneous fat necrosis, and then lead to inflammatory reaction, resulting in local swelling and hard plaque. Oily liquids are often used as an adjunct to progesterone. Oil itself is easy to cause drug malabsorption.^10^ Repeated injection of progesterone can cause accumulation of oily fluid and damage of adipocytes. Patients tend to receive progesterone injection treatment after 1 month effect of local swelling heat pain. Symptoms and signs are not limited to the site of the injection. The increase of serum white blood cell level and CRP level can predict the diagnosis of panniculitis, but it is not completely accurate. Although in our study, patients with panniculitis had higher white blood cell and CRP levels. There are many factors leading to the increase of these two results. Ultrasound and pathology may be necessary.

The author's study also suggests the correlation between oily adjuvant and panniculitis. There are many kinds of injection oils used as excipients of drugs.^11^ Vegetable oil and medium chain oil are the two most common. Our study has not yet confirmed the relationship between medium chain oil and the incidence of panniculitis. Whether medium chain oil as injection oil will increase the incidence of fat necrosis and lead to panniculitis still needs further molecular biological experiments to confirm.

Our study also suggests that there is no relationship between the occurrence of panniculitis and the prognosis of pregnancy. For patients with panniculitis, active and careful treatment is necessary. Contraindications of medication during pregnancy must still be observed. Antibiotics are not necessary if there is no evidence of infection. And the pregnancy status of the patient must be taken into account before receiving antibiotic treatment. Active and effective physical therapy is worth using. The lesion should be thoroughly disinfected first. Liquid dressing can completely isolate the wound from the outside world.^12^ And liquid dressing can play a continuous disinfection function.^13^ Panniculitis often leads to massive exudation. These exudates are mainly due to the body's response to necrotic tissue. Dressings that can absorb a large amount of exudate are suitable for use. In the author's study, all patients achieved satisfactory results, including one patient who did not receive regular treatment. Thorough disinfection and excretion of exudates are the key points of treatment.

Further study is need for the relationship between medium chain oil and panniculitis needs. As the use of progesterone injection is based on hospital procurement, bias may arise.

## 5 CONCLUSION

Progesterone injection may lead to panniculitis, which is rare but may cause serious consequences. Physical therapy can be effective.

## CONFLICT OF INTEREST

The authors declare no potential conflict of interest.

## AUTHOR CONTRIBUTIONS

W.X.: study design. W.X., X.H.: study conduct. W.X.: data interpretation. X.H.: drafting manuscript. W.X. takes responsibility for the integrity of the data analysis. All authors have read and approved the manuscript.

## ETHICS STATEMENT

This study was approved by the institutional review board at The Second Affiliated Hospital of Fujian Medical University (#2020.133). Informed consent was obtained from all individual participants included in the study. Written informed consent was obtained from all the participants in this study. Written informed consent for publication was obtained from all participants.

## Data Availability

The processed data required to reproduce these findings cannot be shared at this time as the data also forms part of an ongoing study.
